# *Helicobacter pylori* infection increases the risk of thyroid nodules in adults of Northwest China

**DOI:** 10.3389/fcimb.2023.1134520

**Published:** 2023-03-31

**Authors:** Jia Di, Zhuang Ge, Qingwei Xie, Danfeng Kong, Sha Liu, Pengwei Wang, Jie Li, Ning Ning, Wei Qu, Rong Guo, Danyan Chang, Jun Zhang, Xiang-hong Zheng

**Affiliations:** ^1^ Department of Nuclear Medicine, The Second Affiliated Hospital of Xi’an Jiaotong University, Xi’an, China; ^2^ Outpatient Department, The Second Affiliated Hospital of Xi’an Jiaotong University, Xi’an, China; ^3^ Department of Gastroenterology, The Second Affiliated Hospital of Xi’an Jiaotong University, Xi’an, China

**Keywords:** *Helicobacter pylori*, thyroid nodules, risk factor, adult, Northwest China

## Abstract

**Background:**

Thyroid nodules (TNs) are very common in the adults of Northwest China. The role of *Helicobacter pylori* (*H. pylori*) infection in TNs is poorly investigated and even with controversial conclusions. Our study aimed at highlighting the relationship between *H. pylori* infection and the risk of TNs.

**Methods:**

9,042 individuals were enrolled with thyroid ultrasonography and ^14^C-urea breath test (^14^C-UBT). Baseline characteristics and relevant covariates were obtained, including basic and laboratory indicators. After applying the exclusion criteria, 8,839 patients were included and divided into 2 groups: a cross-sectional study of single follow-up (*n*=8,711) and a retrospective cohort study of multiple follow-ups for 5 years (*n*=139).

**Results:**

The prevalence of *H. pylori* infection and TNs was 39.58% and 47.94% in the adults of Northwest China, respectively. The prevalence of TNs was significantly higher among *H. pylori*-positive individuals than those without infection (52.55% vs. 44.92%, *p*<0.01). The result of binary logistic regression revealed that the crude odds ratio (OR) was 1.624 (95% CI 1.242~2.123) in Model 1 without adjustment compared to *H. pylori*-negative group, and was also positive in Model 2, 3, and 4 (Model 2: OR=1.731, 95% CI 1.294~2.316; Model 3: OR=2.287, 95% CI 1.633~3.205; Model 4: OR=2.016, 95% CI 1.390~2.922) after the adjustment. The data of 5-year follow-up showed that the annual incidence of TNs was significantly higher in individuals with persistent *H. pylori* infection than non-infected counterparts (all *p*<0.05).

**Conclusions:**

*H. pylori* is an independent risk factor for TNs in the adults of Northwest China.

## 1 Introduction

Thyroid nodules (TNs) are defined as a category of discrete lesions within the thyroid gland by the American Thyroid Association, which can be distinguished from the surrounding thyroid parenchyma in radiography (Haugen et al., 2016). The prevalence of TNs has annually increased, approximately 19%~68% worldwide and an average of 36.9% in China (Bibbins-Domingo et al., 2017; Li et al., 2021). Northwest China is a typically iodine-deficient and economically backward area, and the highest prevalence of TNs in this region reaches 65.6%, which is greater than the average level in China (Chen and Wang, 2016). Ionizing radiation exposure and genetic predisposition are reported to be two major risk factors associated with TNs development in children and adolescents (Bauer, 2019). A combination of dietary habits (iodine intake) and psychosocial factors also affect the occurrence of TNs in the general population (Geng et al., 2023). The traditional risk factors for TNs in Chinese population usually include age, female gender, body mass index (BMI), blood pressure, uric acid, fasting glycemia, etc. (Huang et al., 2022), while novel risk factors for TNs, especially in the adults of Northwest China, are poorly investigated and require further exploration.

Gut microbes play an important role in the development of TNs (Li et al., 2021). As a gram-negative bacterium colonized in the gastric mucosa, *Helicobacter pylori* (*H. pylori*) leads to widespread infection in the world, ranging between 85%~95% in developing countries and fluctuating from 30% to 50% in developed countries (Khoder et al., 2019). Growing studies report that *H. pylori* infection is correlated with the development of multiple thyroid disorders, including TNs (Shen et al., 2013; Figura et al., 2019). However, the data focused on the relationship between *H. pylori* infection and TNs are still inadequate and inconsistent, which contributes to a controversial conclusion (Hu et al., 2020; Wang et al., 2021). Our study aimed to evaluate the association between *H. pylori* infection and the risk of TNs.

## 2 Materials and methods

### 2.1 Study population

**Figure 1:**
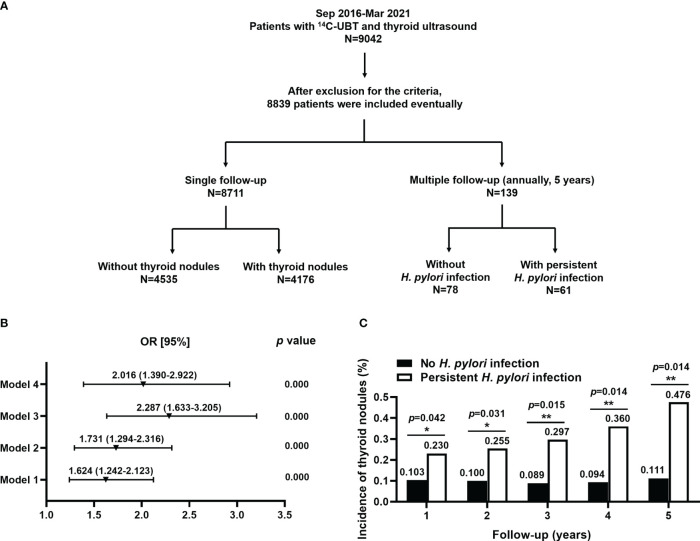
The basic study design and association between (*H*) *pylori* infection and the risk for TNs. **(A)** The basic design flowchart for the study was divided into 2 parts: the single follow-up and multiple follow-ups. A cross-sectional study of single follow-up (*n*=8,711) and a retrospective cohort study of multiple follow-ups (*n*=139) were performed, respectively. **(B)** *H*. *pylori* was an independent risk factor for TNs. Model 1: unadjusted; Model 2: adjusted for age, gender, alcohol, seafood consumption habit, WHR, and SBP; Model 3: model 2 plus an adjustment for smoking, family history of thyroid disease, ALT, AST, HDL-C, and TCH; Model 4: model 3 plus an adjustment for BMI, DBP, FT3, FT4, anti-TPO, Tg, and rT3. **(C)** Persistent *H*. *pylori* infection significantly increased the risk of developing TNs at annual follow-up for 5 years.

Patients admitted to the second affiliated hospital of Xi’an Jiaotong University from September 2016 to March 2021 who underwent ^14^C-UBT and thyroid ultrasonography were included in the study (Shaanxi and Gansu provinces, and Ningxia autonomous region). The individuals were divided into 2 groups: a cross-sectional study of single follow-up and a retrospective cohort study of multiple follow-ups (**Figure 1A**). Patients were excluded on the following criteria: (1) The use of antibiotics, proton pump inhibitors, H_2_ receptor antagonists, and bismuth within 4 weeks before the tests; (2) History of gastric surgery; (3) Allergic to ^14^C-urea; (4) Missing information during the follow-up. The study was performed according to the principles of the Declaration of Helsinki and approved by the ethics committee of the second affiliated hospital of Xi’an Jiaotong University, Xi’an, Shaanxi, China.

### 2.2 Detection of *H. pylori* infection

All participant information was obtained from the clinical record database of the second affiliated hospital of Xi’an Jiaotong University. *H. pylori* infection was diagnosed by ^14^C-UBT, which had been considered the “gold standard” in clinical practice (Aung et al., 2021). In ^14^C-UBT, all participants were required to fast for more than 6 hours before taking ^14^C urea capsule. There was a prohibition against eating and drinking before taking the breath sample after waiting for 30 minutes. The positive result was defined as a standard of over 100 disintegrations per minute per millimole (dpm/mmol), while a range of 0~100 was considered negative.

### 2.3 Definition and classification of TNs

The types of TNs were classified by ultrasonographers with extensive experience in the second affiliated hospital of Xi’an Jiaotong University. TNs were assessed by the American College of Radiology Thyroid Imaging Reporting and Data System (ACR TI-RADS) according to the characteristics of composition, echogenicity, shape, margin, and echogenic foci, which were divided into 5 categories from TR1 (benign) to TR5 (high suspicion of malignancy) (Tessler et al., 2017). To facilitate analysis, TNs had been separately grouped as TR ≤ 2, TR≥4, and TR=3.

### 2.4 Baseline characteristics and relevant covariates

Baseline characteristics included age, gender, body mass index (BMI), waist-hip ratio (WHR), systolic blood pressure (SBP), diastolic blood pressure (DBP), smoking, alcohol, seafood consumption habit, and family history of thyroid disease. BMI was calculated by dividing weight (in kilograms) by the square of height (in meters). WHR was defined as the minimum circumference between the iliac crest and the rib cage. Blood pressure was detected with a mercury sphygmomanometer on the arm after individuals rested for 5 minutes. Hypertension was considered systolic blood pressure (SBP)≥140 mmHg or diastolic blood pressure (DBP)≥90 mmHg or anti-hypertensive treatment.

Relevant covariates involved laboratory indicators related to hepatic-renal functions and thyroid hormones. The fresh blood sample after an overnight fast was obtained from a venipuncture for the following measurements: (1) Hepatic-renal functions: alanine aminotransferase (ALT), aspartate aminotransferase (AST), uric acid (UA), serum creatinine (SCr), total cholesterol (TCH), triglyceride (TG), low-density lipoprotein-cholesterol (LDL-C), high-density lipoprotein-cholesterol (HDL-C), and fasting blood glucose (FBG); (2) Thyroid hormones: thyroid stimulating hormone (TSH), free T3 (FT3), free T4 (FT4), anti-thyroid peroxidase (anti-TPO), anti-thyroglobulin (anti-Tg), thyroglobulin (Tg), thyroid stimulating hormone receptor antibody (TRAb), reverse T3 (rT3), intact parathyroid hormone (iPTH), and thyroid volume. The normal ranges of blood indicators were shown in the supplementary material in details (**Table S1**). The thyroid volume was calculated by measuring left and right lateral lobes with the isthmus by ultrasonography.

### 2.5 Statistical analysis

Statistical analysis was performed by SPSS 22.0 software. The quantitative variables were shown as mean ± standard deviation ( $\bar{\text{X}}\pm \text{SD}$ ) and analyzed by Student’s *t*-test and one-way ANOVA. The qualitative variables were expressed as a percentage (%) with a number of cases and analyzed by the chi-square (χ^2^) test. The crude odds ratios (ORs) were estimated with the binary logistic regression. The assignments of potential risk factors in the logistic regression were presented with numbers 0, 1, 2, and 3 (**Table S2**). The cross-sectional study was performed by a single measurement to explore the association between *H. pylori* and TNs, while multiple assessments were applied to investigate TNs development with *H. pylori* infection in retrospective cohort study. A *p-*value less than 0.05 was considered to be statistically different.

## 3 Results

### 3.1 The basic study design and baseline characteristics of participants with and without TNs

A total of 9,042 individuals were enrolled with ^14^C-UBT and thyroid ultrasonography. After exclusion from the criteria, 8,839 patients were included in the study. A cross-sectional study of single follow-up (*n*=8,711) and a retrospective cohort study of multiple follow-ups (*n*=139) were performed, respectively. In the cross-sectional study, the patients were divided into 2 groups according to the presence (*n*=4,176) or absence (*n*=4,535) of TNs. In the retrospective cohort study, the individuals were also divided into 2 groups: 61 patients with persistent *H. pylori* infection and 78 individuals without infection (**Figure 1A**).

The baseline data showed the characteristics of all enrolled individuals according to the status of TNs. The average prevalence of *H. pylori* infection and TNs was 39.58% and 47.94% in the adults of Northwest China, respectively. The prevalence of TNs was significantly higher in patients with *H. pylori* infection than in those without (52.55% vs. 44.92%, *p*<0.01). The indicators of baseline characteristics were also higher in TNs patients than those without TNs, for example, age, female gender, smoking, alcohol, and seafood consumption habit, as well as family history of thyroid disease. For relevant covariates, the laboratory indicators of hepatic-renal functions and thyroid hormones, also had higher levels in TNs individuals compared to those without TNs, such as ALT, AST, UA, TCH, TSH, FT4, anti-TPO, anti-Tg, Tg, TRAb, and urinary iodine, apart from HDL-C, iPTH, and rT3. Although the *p*-values for indicators BMI, WHR, blood pressure (SBP/DBP), TG, LDL-C, and FT3 were less than 0.01, we did not consider these covariates to be different between the individuals with and without TNs, due to the small effect sizes (*χ^2^/t*). However, the levels of SCr and FBG did not have significant differences between patients with and without TNs (**Table 1**).

**Table 1:** Baseline characteristics of participants with and without TNs.

	With thyroid nodules (N=4176)	Without thyroid nodules (N=4535)	*χ2/t*	*p* value
^14^C-UBT (positive/negative), n (%)	1812/2364, 43.40%	1636/2899, 36.07%	48.66	0.000
Age (years)	52.36 ± 13.82	35.72 ± 13.58	56.65	0.000
Female, n (%)	3060, 73.28%	2492, 54.95%	321.11	0.000
BMI (kg/m^2^)	25.4 ± 1.32	24.3 ± 2.52	25.20	0.000
WHR	0.81 ± 0.19	0.76 ± 0.38	7.67	0.000
SBP (mmHg)	130.67 ± 13.81	125.58 ± 15.86	15.92	0.000
DBP (mmHg)	83.50 ± 12.10	77.36 ± 9.45	26.50	0.000
Smoking, n (%)	1516, 33.43%	1289, 28.42%	61.82	0.000
Alcohol, n (%)	1939, 46.43%	1767, 38.96%	49.61	0.000
Seafood consumption habit, n (%)	311, 7.45%	119, 2.62%	107.78	0.000
Family history of thyroid disease, n (%)	270, 6.47%	21, 0.46%	242.58	0.000
ALT (IU/L)	17.55 ± 10.11	12.17 ± 1.26	35.54	0.000
AST (IU/L)	34.67 ± 2.55	21.64 ± 6.29	124.76	0.000
UA (μmol/L)	322.90 ± 30.30	267.50 ± 9.83	116.64	0.000
SCr (μmol/L)	50.25 ± 11.00	42.29 ± 5.28	9.58	0.149
TCH (mmol/L)	4.74 ± 1.06	3.92 ± 0.63	44.29	0.000
TG (mmol/L)	1.67 ± 0.09	1.47 ± 0.70	18.32	0.000
LDL-C (mmol/L)	3.08 ± 0.68	2.99 ± 0.75	0.61	0.005
HDL-C (mmol/L)	1.25 ± 0.40	1.34 ± 0.36	11.00	0.000
FBG (mmol/L)	5.36 ± 1.59	5.32 ± 0.28	0.10	0.592
TSH (μIU/mL)	8.80 ± 1.68	4.45 ± 0.48	167.12	0.000
FT3 (pmol/L)	5.25 ± 2.64	4.75 ± 0.89	12.03	0.000
FT4 (pmol/L)	20.44 ± 1.09	18.08 ± 1.82	72.65	0.000
Anti-TPO (IU/mL)	73.18 ± 14.50	12.21 ± 2.26	279.50	0.000
Anti-Tg (IU/mL)	186.73 ± 57.61	10.20 ± 0.10	206.35	0.000
Tg (ng/mL)	30.50 ± 7.89	7.45 ± 2.88	183.88	0.000
TRAb (IU/L)	7.94 ± 1.52	1.35 ± 0.31	285.59	0.000
rT3 (ng/dL)	53.00 ± 2.87	58.47 ± 6.63	50.65	0.000
iPTH (pg/mL)	35.63 ± 2.57	40.25 ± 5.53	50.64	0.000
Urinary iodine (µg/L)	303.34 ± 158.56	215.51 ± 105.48	30.66	0.000
Thyroid volume (mL)	30.06 ± 3.11	21.97 ± 8.64	57.19	0.000

Data were expressed as mean ± standard deviation or sample size (n, %). ^*^p<0.05 and ^**^p<0.01 were considered statistically significant. ^14^C-UBT, ^14^C-urea breath test; BMI, body mass index; WHR, waist-hip ratio; SBP, systolic blood pressure; DBP, diastolic blood pressure; ALT, alanine transaminase; AST, aspartate aminotransferase; UA, uric acid; SCr, serum creatinine; TCH, total cholesterol; TG, triglycerides; LDL-C, high-density lipoprotein-cholesterol; HDL-C, low-density lipoprotein-cholesterol; FBG, fasting blood glucose; TSH, thyroid stimulating hormone; FT3, free T3; FT4, free T4; Anti-TPO, anti-thyroid peroxidase; Anti-Tg, anti-thyroglobulin; Tg, thyroglobulin; TRAb, thyroid stimulating hormone receptor antibody; rT3, reverse T3; iPTH, intact parathyroid hormone.

### 3.2 The association between *H. pylori* infection and the risk of TNs

The prevalence of TNs between positive and negative *H. pylori* patients was calculated, and the relevant covariates were also analyzed (**Table 1**). The average prevalence of TNs was 47.94% in the northwestern Chinese adults detected by ultrasonography. The prevalence of TNs was significantly higher in *H. pylori*-positive individuals than those without infection (52.55% vs. 44.92%, *p*<0.01). In addition, significant prevalence of TNs was also observed in basic indicators, for example, advanced age, female gender, smoking, alcohol, seafood consumption habit, and family history of thyroid disease (all *p*<0.01). Moreover, we also found an increased prevalence of TNs in laboratory indicators, such as ALT, AST, UA, TCH, TSH, FT4, anti-TPO, anti-Tg, Tg, TRAb, urinary iodine, and thyroid volume (all *p*<0.01). However, there was no significant difference in SCr and FBG between patients with and without TNs (*p*=0.15 and *p*=0.59). Also, it was noted that rT3 and iPTH were all decreased in subjects with TNs than those without TNs (all *p*<0.01).

TNs were divided into 5 types according to ACR TI-RADS (TR1~5). We combined these types into 3 clusters, namely TR ≤ 2, TR=3, and TR≥4. There were significant differences in *H. pylori* infection among 3 clusters of TNs (χ^2 =^ 152.47, *p*<0.01, **Table 2**). The proportion of *H. pylori* infection in the TR≥4 group was considerably higher than that in the TR=3 group (Bonferroni, χ^2 =^ 76.46, *p*<0.01), but slightly lower compared with the TR ≤ 2 group (Bonferroni, χ^2 =^ 124.33, *p*<0.01). A binary logistic regression was performed, which obtained 4 models with odds ratios (ORs, **Figure 1B**). As shown in Model 1, the unadjusted OR for the association between *H. pylori* infection and TNs was 1.624 (95% CI 1.242~2.123, *p*<0.01). Model 2 indicated that *H. pylori* infection was related to an increased risk of TNs with an OR of 1.731 (95% CI 1.294~2.316, *p*<0.01) after an adjustment for age, gender, alcohol, seafood consumption habit, WHR, and SBP. Model 3 revealed a significant positive correlation (OR=2.287, 95% CI 1.633~3.205, *p*<0.01) adjusted for additional relevant covariates, including smoking, family history of thyroid disease, ALT, AST, HDL-C, and TCH. On the basis of Model 3, Model 4 indicated that *H. pylori* was associated with TNs but with a slightly lower risk (OR=2.016, 95% CI 1.390~2.922, *p*<0.01) than that in Model 3 after an adjustment for BMI, DBP, FT3, FT4, anti-TPO, Tg, and rT3 (**Table 2**).

**Table 2:** The association between *H. pylori* infection and risk of TNs.

^14^C-UBT	Without TNs	The classification of TN types, n (%)			Model 1	Model 2	Model 3	Model 4
		TI-RADS ≤ 2	TI-RADS=3	TI-RADS≥4	OR (95% CI)	OR (95% CI)	OR (95% CI)	OR (95% CI)
*H. pylori* (negative), n (%)	2899, 33.28%	660, 7.58%	309, 3.55%	1395, 16.01%	1.000 (reference)	1.000 (reference)	1.000 (reference)	1.000 (reference)
*H. pylori* (positive), n (%)	1636, 18.78%	743, 8.53%	349, 4.01%	720, 8.27%	1.624 (1.242~2.123)	1.731 (1.294~2.316)	2.287 (1.633~3.205)	2.016 (1.390~2.922)
*χ^2^/p* value	–	152.47, 0.000^**^			0.000^**^	0.000^**^	0.000^**^	0.000^**^

Model 1: unadjusted;

Model 2: adjusted for age, gender, alcohol, seafood consumption habit, WHR, and SBP;

Model 3: model 2 + adjusted for smoking, family history of thyroid disease, ALT, AST, HDL-C, and TCH;

Model 4: model 3 + adjusted for BMI, DBP, FT3, FT4, anti-TPO, Tg, and rT3.

^**^p<0.01 is considered statistically significant. ^14^C-UBT, ^14^C-urea breath test; WHR, waist-hip ratio; SBP, systolic blood pressure; ALT, alanine transaminase; AST, aspartate aminotransferase; HDL-C, low-density lipoprotein-cholesterol; TCH, total cholesterol; BMI, body mass index; DBP, diastolic blood pressure; FT3, free T3; FT4, free T4; Anti-TPO, anti-thyroid peroxidase; Tg, thyroglobulin; rT3, reverse T3.

### 3.3 The association between persistent *H. pylori* infection and the risk of developing TN types at annual follow-up for 5 years

A total of 139 patients had been followed-up by ^14^C-UBT and thyroid ultrasonography for 5 years. Individuals with annually positive ^14^C-UBT were considered persistent *H. pylori* infection and TN types were classified by ultrasonography according to ACR TI-RAD. The annual incidences of TNs were all significantly higher in patients with persistent *H. pylori* infection (all *p*<0.05) than those without infection at 5-year follow-ups (**Figure 1C**). There were 14 and 8 new cases with TNs existing in individuals with persistent infection of *H. pylori* and without infection in the 1^st^ year of the follow-up, respectively. After 5 years, a total of 80 new cases developed TNs during the follow-up, including 57 cases with persistent *H. pylori* infection and 23 cases without infection (**Table 3**).

**Table 3:** New cases of patients with TNs at annual follow-up for 5 years.

H. pylori	Number	Time (years)				
		1	2	3	4	5
No *H. pylori* infection	Total	78	60	45	32	18
	New case	8	6	4	3	2
Persistent *H. pylori* infection	Total	61	51	37	25	21
	New case	14	13	11	9	10

The association between persistent *H. pylori* infection and the development of TN types (TR ≤ 2, TR=3, and TR≥4) had been investigated in the 1^st^ year of 5-year follow-up (**Table 4**). For TNs individuals with *H. pylori* infection, the indicators including WHR, alcohol, TG, rT3, and iPTH, were all significantly higher than those without infection (all *p*<0.05). It was interesting to note that WHR, TG, and rT3 were significantly greater in positive patients than their counterparts in the TR≥4 group (all *p*<0.01). However, we only observed a slight difference of UA in the TR≥4 group (*p*=0.047), and this difference did not occur in the TR ≤ 2 and TR=3 groups. For other remaining indicators, no differences were observed, regardless of TN types.

**Table 4:** The association between persistent *H. pylori* infection and risk of developing TN types in the 1^st^ year of 5-year follow-up.

Variables	Total			TI-RADS ≤ 2			TI-RADS=3			TI-RADS≥4		
	*H. pylori* (+)	*H. pylori* (-)	*p* value	*H. pylori* (+)	*H. pylori* (-)	*p* value	*H. pylori* (+)	*H. pylori* (-)	*p* value	*H. pylori* (+)	*H. pylori* (-)	*p* value
^14^C-UBT, n (%)	61, 43.88%	78, 56.12%	–	17, 34.69%	32, 65.31%	–	8, 34.78%	15, 65.22%	–	36, 53.73%	31, 46.27%	–
Age (years)	52.65 ± 13.63	52.79 ± 13.40	0.952	52.49 ± 13.68	52.64 ± 13.72	0.971	52.74 ± 13.55	52.91 ± 12.84	0.977	52.58 ± 13.70	52.82 ± 13.67	0.943
Female, n (%)	44, 31.65%	58, 41.73%	0.768	12, 24.49%	24, 48.98%	0.739	8, 34.78%	11, 47.83%	0.108	24, 35.82%	23, 34.33%	0.502
BMI (kg/m^2^)	25.51 ± 1.21	25.22 ± 1.09	0.140	25.46 ± 1.18	25.09 ± 0.96	0.242	25.55 ± 1.22	25.23 ± 0.89	0.478	25.56 ± 1.20	25.31 ± 1.11	0.382
WHR	0.82 ± 0.10	0.78 ± 0.08	0.010^*^	0.79 ± 0.21	0.76 ± 0.08	0.475	0.80 ± 0.07	0.78 ± 0.04	0.389	0.84 ± 0.09	0.78 ± 0.08	0.006^**^
SBP (mmHg)	131.11 ± 15.86	130.59 ± 15.86	0.848	131.19 ± 16.14	130.96 ± 12.04	0.955	131.20 ± 15.10	130.58 ± 16.17	0.928	130.97 ± 12.08	129.70 ± 16.01	0.713
DBP (mmHg)	83.55 ± 11.54	83.37 ± 11.45	0.927	83.66 ± 11.77	83.45 ± 11.58	0.952	83.48 ± 11.60	83.31 ± 10.93	0.973	83.52 ± 11.65	83.13 ± 10.89	0.888
Smoking, n (%)	19, 13.67%	23, 16.55%	0.832	7, 14.29%	11, 7.91%	0.638	4, 17.39%	5, 21.74%	0.436	8, 11.94%	7, 10.45%	0.975
Alcohol, n (%)	24, 17.27%	18, 12.95%	0.038^*^	6, 12.24%	8, 16.33%	0.448	4, 17.39%	4, 17.39%	0.263	15, 22.39%	5, 7.46%	0.023^*^
Seafood consumption habit, n (%)	8, 5.76%	7, 5.04%	0.435	2, 4.08%	2, 4.08%	0.502	2, 8.70%	1, 4.35%	0.214	4, 5.97%	4, 5.97%	0.821
ALT (IU/L)	17.95 ± 10.12	17.64 ± 10.03	0.857	17.55 ± 10.25	17.34 ± 10.69	0.947	18.20 ± 10.36	18.69 ± 9.18	0.908	18.32 ± 10.94	17.10 ± 10.24	0.639
AST (IU/L)	34.74 ± 2.29	34.53 ± 2.35	0.597	34.64 ± 1.89	34.20 ± 2.41	0.486	34.93 ± 2.26	34.44 ± 2.30	0.630	34.64 ± 2.29	34.76 ± 1.85	0.813
UA (μmol/L)	319.81 ± 30.68	315.00 ± 30.63	0.360	313.01 ± 28.39	310.64 ± 33.12	0.795	307.32 ± 32.64	312.73 ± 31.90	0.705	332.09 ± 30.34	306.81 ± 31.25	0.047^*^
SCr (μmol/L)	50.43 ± 11.02	50.11 ± 11.23	0.866	50.12 ± 11.16	49.13 ± 11.34	0.771	50.52 ± 11.22	49.74 ± 11.04	0.874	50.39 ± 11.15	50.34 ± 9.93	0.985
TCH (mmol/L)	4.70 ± 1.04	4.54 ± 1.28	0.418	4.74 ± 1.03	4.64 ± 1.07	0.752	4.66 ± 1.07	4.81 ± 1.05	0.749	4.71 ± 1.01	4.38 ± 1.05	0.195
TG (mmol/L)	1.47 ± 0.11	1.42 ± 0.07	0.003^**^	1.46 ± 0.10	1.44 ± 0.16	0.594	1.50 ± 0.14	1.48 ± 0.01	0.580	1.47 ± 0.12	1.36 ± 0.08	0.000^**^
LDL-C (mmol/L)	2.76 ± 0.73	2.72 ± 0.75	0.752	2.74 ± 0.72	2.69 ± 0.69	0.816	2.83 ± 0.88	2.65 ± 0.70	0.596	2.76 ± 0.74	2.73 ± 0.73	0.868
HDL-C (mmol/L)	1.36 ± 0.35	1.46 ± 0.25	0.052	1.32 ± 0.36	1.43 ± 0.28	0.242	1.34 ± 0.38	1.45 ± 0.26	0.420	1.39 ± 0.31	1.47 ± 0.20	0.208
FBG (mmol/L)	5.35 ± 1.60	5.34 ± 1.56	0.971	5.30 ± 1.61	5.32 ± 1.63	0.967	5.35 ± 1.57	5.36 ± 1.68	0.989	5.36 ± 1.64	5.32 ± 1.62	0.921
TSH (μIU/mL)	8.76 ± 1.66	8.71 ± 1.47	0.851	8.61 ± 1.73	8.67 ± 1.71	0.908	8.97 ± 1.72	9.02 ± 1.73	0.948	9.06 ± 1.71	8.77 ± 1.69	0.489
FT3 (pmol/L)	5.23 ± 1.60	5.13 ± 1.52	0.707	5.25 ± 1.69	5.11 ± 1.54	0.771	5.28 ± 1.68	5.18 ± 1.39	0.880	5.22 ± 1.58	5.10 ± 1.52	0.753
FT4 (pmol/L)	20.25 ± 1.05	20.33 ± 1.35	0.695	20.34 ± 1.83	20.26 ± 1.36	0.863	20.23 ± 1.02	20.56 ± 1.11	0.484	20.25 ± 1.06	20.13 ± 1.05	0.644
Anti-TPO (IU/mL)	75.24 ± 14.53	73.83 ± 14.40	0.569	73.83 ± 14.90	74.39 ± 14.79	0.900	78.84 ± 14.93	71.41 ± 14.17	0.253	69.82 ± 13.97	75.59 ± 14.64	0.104
Anti-Tg (IU/mL)	184.65 ± 57.32	187.44 ± 56.77	0.775	184.44 ± 59.01	187.47 ± 58.11	0.864	187.37 ± 58.76	190.24 ± 58.96	0.912	180.63 ± 58.01	185.67 ± 57.75	0.723
Tg (ng/mL)	30.09 ± 5.84	30.69 ± 5.80	0.547	29.69 ± 6.20	30.38 ± 6.09	0.709	32.53 ± 6.05	30.75 ± 6.14	0.513	31.32 ± 5.89	30.48 ± 5.45	0.549
TRAb (IU/L)	7.83 ± 1.55	7.94 ± 1.52	0.675	7.94 ± 1.61	8.25 ± 1.32	0.472	8.49 ± 1.61	8.04 ± 1.25	0.520	7.27 ± 1.56	7.82 ± 1.55	0.154
rT3 (ng/dL)	52.12 ± 2.60	53.07 ± 2.54	0.032^*^	52.67 ± 2.69	53.21 ± 2.64	0.502	52.61 ± 2.64	53.52 ± 2.49	0.424	50.83 ± 2.39	52.82 ± 2.60	0.002^*^
iPTH (pg/mL)	35.64 ± 2.47	34.51 ± 2.55	0.010^*^	35.63 ± 2.48	34.46 ± 2.50	0.125	35.36 ± 2.60	34.21 ± 2.57	0.320	35.95 ± 2.38	34.64 ± 2.44	0.030^*^
Thyroid volume (mL)	30.50 ± 3.60	30.46 ± 3.51	0.948	30.89 ± 3.74	30.85 ± 3.69	0.971	31.22 ± 3.70	30.37 ± 3.75	0.608	29.87 ± 3.69	30.29 ± 3.67	0.643
Family history of thyroid disease, n (%)	6, 4.32%	5, 3.60%	0.458	2, 4.08%	2, 4.08%	0.502	2, 8.70%	1, 4.35%	0.214	2, 2.99%	2, 2.99%	0.877

Data were expressed as mean ± standard deviation or sample size (n, %). ^*^p<0.05 and ^**^p<0.01 are considered statistically significant. ^14^C-UBT, ^14^C-urea breath test; BMI, body mass index; WHR, waist-hip ratio; SBP, systolic blood pressure; DBP, diastolic blood pressure; ALT, alanine transaminase; AST, aspartate aminotransferase; UA, uric acid; SCr, serum creatinine; TCH, total cholesterol; TG, triglycerides; LDL-C, high-density lipoprotein-cholesterol; HDL-C, low-density lipoprotein-cholesterol; FBG, fasting blood glucose; TSH, thyroid stimulating hormone; FT3, free T3; FT4, free T4; Anti-TPO, anti-thyroid peroxidase; Anti-Tg, anti-thyroglobulin; Tg, thyroglobulin; TRAb, thyroid stimulating hormone receptor antibody; rT3, reverse T3; iPTH, intact parathyroid hormone.

## 4 Discussion

Our study demonstrated that: (1) *H. pylori* infection and TNs were both common in the adults of Northwest China, and the prevalence of TNs was higher in *H. pylori*-positive individuals than those without infection; (2) *H. pylori* was an independent risk factor for TNs whether or not adjusting for relevant covariates; (3) The development of TNs had been detected in northwestern Chinese adults with persistent infection of *H. pylori* in multiple follow-ups for 5 years.

Northwest China is a naturally iodine-deficient and economically backward region, contributing to a high prevalence of TNs. Except for traditional risk factors, studies concerning novel risk factors, such as *H. pylori*, has not been fully investigated. In our cross-sectional study, the result of logistic regression revealed a correlation between *H. pylori* infection and TNs independent of other relevant covariates. The study reported that *H. pylori* infection had been positively associated with the presence of TNs, which was consistent with our results (Shen et al., 2013). Moreover, a case-control study including 370 cases indicated that *H. pylori* infection was significantly higher in patients with benign TNs than in the control group (Bakhshipour et al., 2022). However, there was also an opposite view that the association between *H. pylori* infection and TNs risk was a lack of sufficient evidence (Wang et al., 2021). The reasons for this discrepancy among different cohorts remained unclear. It might be originated from the biases of selection, information, and confounding. For selection bias, the representative samples of the target population were different, such as adults, adolescents, children, and infants. Information bias was usually caused by a lack of accurate measurements of the variables, such as different methods for detecting *H. pylori* and TNs, so the standardized method was important at the baseline. Confounding bias prevented study conclusions from reflecting true associations, and the most common confounding factors in the cohort were gender and age.

Although there is no definite consensus on the association between *H. pylori* infection and TNs risk, the mechanism has not been fully investigated. It has been reported that molecular mimicry is the integrated mechanism of autoimmune thyroid disorder caused by *H. pylori* (Cuan-Baltazar and Soto-Vega, 2020). There is a cross-reactivity between *H. pylori* antibodies and thyroid follicles, such as cytotoxin associated gene A, which has a nucleotide sequence similar to thyroid peroxidase (Bassi et al., 2010). Increased levels of inflammatory cytokines and accelerated lymphocytic infiltrations into thyroid follicles lead to thyroid inflammation and autoimmune thyroid disease, especially TNs (Bassi et al., 2010; Figura et al., 2019). Although molecular mimicry is the mainstream hypothesis, it cannot fully explain the mechanism of TNs development. The scholars (Zhang et al., 2019; Wang et al., 2021) have proposed a complementary mechanism for dysbiosis, pointing out the induction of thyroid cancer and TNs are both associated with imbalanced composition of gut microbiome, such as an increasing proportion of *H. pylori*.

In addition to *H. pylori*, whether iodine intake induced TNs remained controversial. The opposite conclusion might be contributed to autoimmune thyroiditis caused by unusual iodine intake (too high or too low), which promoted chronic cell proliferation and differentiation. It was interesting to note that proteins, such as anti-TPO, anti-Tg, TRAb, and Tg, increased significantly in TNs individuals. According to the reports (Krátký et al., 2018), both anti-TPO and anti-Tg elevations showed a positive correlation with TNs development. As a TSH receptor, a growing TRAb probably led to Grave’s disease with functional or non-functional TNs (Aydin et al., 2020). In addition, as a tumor marker of the thyroid gland, elevated Tg was often accompanied by TNs, particularly in benign nodules (Hu et al., 2019; Murphy and Gupta, 2020). However, we observed that rT3 decreased in TNs patients, which were opposed to alterations in other thyroid indicators. The decline in rT3 was attributed to the decrease in rT3 production from T4, or the increase in the clearance of rT3 to diiodothyronine (Rhee and Kalim, 2018). Thus, it was not difficult to understand a reduction of rT3 in our results, which was compatible with an increase in FT4.

The development of TNs had been detected in Northwest Chinese adults with persistent infection of *H. pylori* in multiple follow-ups for 5 years. Higher levels of WHR and TG were observed in *H. pylori*-positive individuals with TNs than those without infection, which was more prominent in the TR≥4 group. It was also reported that *H. pylori* resulted in a high WHR (over 0.85 and 0.90 in women and men) and TG, which promoted an elevated risk of TNs (Sharma et al., 2016; Song et al., 2018; Murphy and Gupta, 2020; Jiang et al., 2022). The mechanism of TG inducing TNs might be related to insulin resistance increasing expanded thyroid proliferation and nodular formation (Yasar et al., 2011). We also found that persistent *H. pylori* infection contributed to the disruption of thyroid hormones, with a decrease in rT3 and an increase in iPTH, especially in the type of TR≥4. The opposing levels of rT3 and iPTH appeared in TNs groups, and its mechanism was speculated as the long-term infection of *H. pylori* causing irreversible dysfunctions in thyroid and parathyroid glands.

There were a few limitations in our study. Firstly, our study, including cross-sectional and cohort studies, all involved a single-center population. A multicenter data could be better to explain the association between *H. pylori* and TNs risk. Secondly, there might be a potential observer variation in the TI-RADS grading of TNs by different ultrasonographers. Thirdly, the impact of *H. pylori* eradication therapy on TNs development remained unclear, which was a part deserving of further study. Finally, there were only 139 individuals initially in the retrospective cohort for a 5-year follow-up, while it fell to a total of 39 after the whole follow-up. Thus, we were prevented from analyzing data after the entire follow-up due to insufficient individuals, but better to complete the analysis in the 1^st^ year.

## Data availability statement

The raw data supporting the conclusions of this article will be made available by the authors, without undue reservation.

## Ethics statement

The studies involving human participants were reviewed and approved by the ethics committee of the second affiliated hospital of Xi’an Jiaotong University, Xi’an, Shaanxi, China. The patients/participants provided their written informed consent to participate in this study.

## Author contributions

JD analyzed the data and wrote the whole manuscript. X-HZ provided the idea and designed the study. ZG, QX, DK, and SL collected the clinical data. RG and NN made the questionnaire. DC, PW, and JL assisted in the analysis of data. WQ and JZ assisted in revising the article. All authors contributed to the article and approved the submitted version.

## References

[B1] Aung W. P. Aye T. T. Aye K. S. Kyaw A. M. M. (2021). Levofloxacin-based *Helicobacter pylori* eradication in chronic dyspepsia. GastroHep. 3, 394–400. doi: 10.1002/ygh2.478

[B2] Aydin C. Bader H. Cuhaci N. Ozdemir D. Ersoy R. Cakir B.. (2020). Thyrotrophin receptor antibody is not associated with thyroid cancer in patients with toxic nodular and multinodular goiter. Ankara. Medical. J. 20, 234–241. doi: 10.5505/amj.2020.79553

[B3] Bakhshipour A. Amirian M. Heidari Z. (2022). Association of *Helicobacter pylori* infection with papillary thyroid carcinoma: A case-control study. Int. J. Cancer Manage. 15, e118031. doi: 10.5812/ijcm-118031

[B4] Bassi V. Santinelli C. Iengo A. Romano C. (2010). Identification of a correlation between *Helicobacter pylori* infection and graves’ disease. Helicobacter 15, 558–562. doi: 10.1111/j.1523-5378.2010.00802.x 21073613

[B5] Bauer A. J. (2019). Thyroid nodules in children and adolescents. Curr. Opin. Endocrinol. Diabetes Obes. 26, 266–274. doi: 10.1097/MED.0000000000000495 31361657

[B6] Bibbins-Domingo K. Grossman D. C. Curry S. J. Barry M. J. Davidson K. W. Doubeni C. A.. (2017). Screening for thyroid cancer: US preventive services task force recommendation statement. JAMA 317, 1882–1887. doi: 10.1001/jama.2017.4011 28492905

[B7] Chen P. Wang M. (2016). Investigation on the incidence of thyroid disease among 16929 medical workers in western China. J. Baotou Med. Coll. 32, 9–11. doi: 10.16833/j.cnki.jbmc.2016.01.006

[B8] Cuan-Baltazar Y. Soto-Vega E. (2020). Microorganisms associated to thyroid autoimmunity. Autoimmun. Rev. 19, 2614. doi: 10.1016/j.autrev.2020.102614 32663624

[B9] Figura N. Di Cairano G. Moretti E. Iacoponi F. Santucci A. Bernardini G.. (2019). *Helicobacter pylori* infection and autoimmune thyroid diseases: The role of virulent strains. Antibiotics (Basel) 9, 12. doi: 10.3390/antibiotics9010012 31906000 PMC7167994

[B10] Geng D. Zhou Y. Su G. Y. Si Y. Shen M. P. Xu X. Q.. (2023). Influence of sex, age and thyroid function indices on dual-energy computed tomography-derived quantitative parameters of thyroid in patients with or without hashimoto’s thyroiditis. BMC Med. Imaging 23, 25. doi: 10.1186/s12880-023-00983-x 36740672 PMC9901076

[B11] Haugen B. R. Alexander E. K. Bible K. C. Doherty G. M. Mandel S. J. Nikiforov Y. E.. (2016). 2015 American thyroid association management guidelines for adult patients with thyroid nodules and differentiated thyroid cancer: the American thyroid association guidelines task force on thyroid nodules and differentiated thyroid cancer. Thyroid 26, 1–133. doi: 10.1089/thy.2015.0020 26462967 PMC4739132

[B12] Hu Y. Li N. Jiang P. Cheng L. Ding B. Liu X. M.. (2019). Elevated thyroglobulin level is associated with dysfunction of regulatory T cells in patients with thyroid nodules. Endocr. Connect. 8, 309–317. doi: 10.1530/ec-18-0545 30822273 PMC6432874

[B13] Hu L. Li T. Yin X. L. Zou Y. (2020). An analysis of the correlation between thyroid nodules and metabolic syndrome. Endocr. Connect. 9, 933–938. doi: 10.1530/ec-20-0398 33006954 PMC7583134

[B14] Huang Y. Li Z. Yang K. Zhang L. Wei C. Yang P.. (2022). The association of uric acid with the development of thyroid nodules: a retrospective cohort study. BMC Endocr. Disord. 22, 197. doi: 10.1186/s12902-022-01119-y 35941598 PMC9358884

[B15] Jiang Y. Huang L. Zhou L. (2022). Association between obesity and *Helicobacter pylori* infection. Nutr. Clin. Metab. 36, 210–216. doi: 10.1016/j.nupar.2022.07.003

[B16] Khoder G. Muhammad J. S. Mahmoud I. Soliman S. S. M. Burucoa C. (2019). Prevalence of *Helicobacter pylori* and its associated factors among healthy asymptomatic residents in the united Arab Emirates. Pathogens 8, 44. doi: 10.3390/pathogens8020044 30939800 PMC6632043

[B17] Krátký J. Ježková J. Kosák M. Vítková H. Bartáková J. Mráz M.. (2018). Positive antithyroid antibodies and nonsuppressed TSH are associated with thyroid cancer: A retrospective cross-sectional study. Int. J. Endocrinol. 2018, 9793850. doi: 10.1155/2018/9793850 30258461 PMC6146563

[B18] Li Y. Jin C. Li J. Tong M. Wang M. Huang J.. (2021). Prevalence of thyroid nodules in China: A health examination cohort-based study. Front. Endocrinol. (Lausanne). 12. doi: 10.3389/fendo.2021.676144 PMC8188053 34122350

[B19] Li A. Li T. Gao X. Yan H. Chen J. Huang M.. (2021). Gut microbiome alterations in patients with thyroid nodules. Front. Cell. Infect. 11. doi: 10.3389/fcimb.2021.64396 PMC8005713 33791245

[B20] Murphy C. Gupta A. (2020). MON-458 elevated thyroglobulin level in benign thyroid nodule. J. Endocr. Soc. (Supplement 1), MON–458. doi: 10.1210/jendso/bvaa046.1436

[B21] Rhee C. M. Kalim S. (2018). “Chapter 27 - thyroid status in chronic renal failure patients,” in Textbook of nephro-endocrinology, 2nd ed. Eds. Singh A. K. Williams G. H. (Academic Press), 477–492.

[B22] Sharma S. Batsis J. A. Coutinho T. Somers V. K. Hodge D. O. Carter R. E.. (2016). “Normal-weight central obesity and mortality risk in older adults with coronary artery disease,” in Mayo Clinic proceedings (Elsevier), 343–351. 10.1016/j.mayocp.2015.12.007 26860580

[B23] Shen Z. Qin Y.e. Liu Y. Lu Y. Munker S. Chen L.. (2013). *Helicobacter pylori* infection is associated with the presence of thyroid nodules in the euthyroid population. PloS One 8, e80042. doi: 10.1371/journal.pone.0080042 24244604 PMC3823768

[B24] Song B. Zuo Z. Tan J. Guo J. Teng W. Lu Y.. (2018). Association of thyroid nodules with adiposity: A community-based cross-sectional study in China. BMC Endocr. Disord. 18, 3. doi: 10.1186/s12902-018-0232-8 29374470 PMC5787304

[B25] Tessler F. N. Middleton W. D. Grant E. G. Hoang J. K. Berland L. L. Teefey S. A.. (2017). ACR thyroid imaging, reporting and data system (TI-RADS): white paper of the ACR TI-RADS committee. JACR 14, 587–595. doi: 10.1016/j.jacr.2017.01.046 28372962

[B26] Wang X. S. Xu X. H. Jiang G. Ling Y. H. Ye T. T. Zhao Y. W.. (2021). Lack of association between *Helicobacter pylori* infection and the risk of thyroid nodule types: a multicenter case-control study in China. Front. Cell. Infect. 1245. doi: 10.3389/fcimb.2021.766427 PMC8713074 34970506

[B27] Yasar H. Y. Ertuğrul O. Ertuğrul B. Ertuğrul D. Sahin M. (2011). Insulin resistance in nodular thyroid disease. Endocr. Res. 36, 167–174. doi: 10.3109/07435800.2011.593011 21973236

[B28] Zhang J. Zhang F. Zhao C. Xu Q. Liang C. Yang Y.. (2019). Dysbiosis of the gut microbiome is associated with thyroid cancer and thyroid nodules and correlated with clinical index of thyroid function. Endocrine 64, 564–574. doi: 10.1007/s12020-018-1831-x 30584647

