# Supplementary material for: Helicobacter pylori infection increases the risk of thyroid nodules in adults of Northwest China
Source: Front Cell Infect Microbiol. 2023 Mar 31;13:1134520. doi: 10.3389/fcimb.2023.1134520 (PMC10102366; doi:10.3389/fcimb.2023.1134520)
Supplement: Supplementary file 1 [file Table_1.docx]

| **Indicator** | **Normal range (unit)** |
| --- | --- |
| ALT | 7~40 (IU/L) |
| AST | 13~35 (IU/L) |
| UA | 155~357 (μmol/L) |
| TCH | 2.8~5.2 (mmol/L) |
| TG | 0.56~1.70 (mmol/L) |
| LDL-C | 1.04~1.55 (mmol/L) |
| HDL-C | 2.1~3.1 (mmol/L) |
| TSH | 0.27~4.20 (μIU/mL) |
| FT3 | 3.1~6.6 (pmol/L) |
| FT4 | 12~22 (pmol/L) |
| Anti-TPO | 0~34 (IU/mL) |
| Anti-Tg | 0~115 (IU/mL) |
| Tg | 3.5~77.0 (ng/mL) |
| TRAb | 0.00~1.75 (IU/L) |
| rT3 | 35~95 (ng/dL) |
| iPTH | 15~65 (pg/mL) |

Supplementary Material

*Helicobacter pylori* infection increases the risk of thyroid nodules in adults of Northwest China

**Jia Di*****, Zhuang Ge, Qingwei Xie, Danfeng Kong, Sha Liu, Pengwei Wang, Jie Li, Ning Ning, Wei Qu, Rong Guo, Danyan Chang, Jun Zhang, Xiang-hong Zheng***

*** Correspondence:** Corresponding Author: Xiang-hong Zheng, 2275630208@qq.com

# Supplementary Tables

**Table S1. Normal ranges of laboratory indicators for relevant covariates.**

ALT, alanine transaminase; AST, aspartate aminotransferase; UA, uric acid; TCH, total cholesterol; TG, triglycerides; LDL-C, high-density lipoprotein-cholesterol; HDL-C, low-density lipoprotein-cholesterol; TSH, thyroid stimulating hormone; FT3, free T3; FT4: free T4; Anti-TPO, anti-thyroid peroxidase; Anti-Tg, anti-thyroglobulin; Tg, thyroglobulin; TRAb, thyroid stimulating hormone receptor antibody; rT3, reverse T3; iPTH, intact parathyroid hormone.

**Table S2. The assignments for TNs indicators.**

| **Indicators** | **Variables** | **Assignments** |
| --- | --- | --- |
| ^14^C-UBT, n (%) | X1 | Positive=1, negative=0 |
| Gender | X2 | Male=1, female=0 |
| Smoking, n (%) | X3 | Smoking=1, non-smoking=0 |
| Alcohol, n (%) | X4 | Alcohol=1, non-alcohol=0 |
| Seafood consumption habit, n (%) | X5 | Seafood consumption habit=1, non-seafood consumption habit=0 |
| Family history, n (%) | X6 | Family history=1, non-family history=0 |
| Age | X7 | ≤18=0, 19~25=1, 26~65=2, ≥66=3 |
| BMI | X8 | ＜18.5=0, 18.5~24.9=1, ≥25.0=2 |
| WHR | X9 | Male:≤0.95=0, 0.96~0.99=1, ≥1.00=2  Female:≤0.80=0, 0.81~0.85=1, ≥0.86=2 |
| SBP | X10 | ＜140=0, ≥140=1 |
| DBP | X11 | ＜90=0, ≥90=1 |
| ALT | X12 | ＜7=0, 7~40=1, ＞40=2 |
| AST | X13 | ＜13=0, 13~35=1, ＞35=2 |
| UA | X14 | ＜155=0, 155~357=1, ＞357=2 |
| TCH | X15 | ＜2.8=0, 2.8~5.2=1, ＞5.2=2 |
| TG | X16 | ＜0.56=0, 0.56~1.70=1, ＞1.70=2 |
| LDL-C | X17 | ＜2.1=0, 2.1~3.1=1, ＞3.1=2 |
| HDL-C | X18 | ＜1.04=0, 1.04~1.55=1, ＞1.55=2 |
| TSH | X19 | ＜0.27=0, 0.27~4.2=1, ＞4.2=2 |
| FT3 | X20 | ＜3.1=0, 3.1~6.8=1, ＞6.8=2 |
| FT4 | X21 | ＜12=0, 12~22=1, ＞22=2 |
| Anti-TPO | X22 | ≤34=0, ＞34=1 |
| Anti-Tg | X23 | ≤115=0, ＞115=1 |
| Tg | X24 | ＜3.5=0, 3.5~77.0=1, ＞77.0=2 |
| TRAb | X25 | ≤1.75=0, ＞1.75=1 |
| rT3 | X26 | ＜35=0, 35~95=1, ＞95=2 |
| iPTH | X27 | ＜15=0, 15~65=1, ＞65=2 |
| Thyroid volume | X28 | ＜20=0, 20~30=1, ＞30=2 |

^14^C-UBT, ^14^C-urea breath test; BMI, body mass index; WHR, waist-hip ratio; SBP, systolic blood pressure; DBP, diastolic blood pressure; ALT, alanine transaminase; AST, aspartate aminotransferase; UA, uric acid; TCH, total cholesterol; TG, triglycerides; LDL-C, high-density lipoprotein-cholesterol; HDL-C, low-density lipoprotein-cholesterol; TSH, thyroid stimulating hormone; FT3, free T3; FT4: free T4; Anti-TPO, anti-thyroid peroxidase; Anti-Tg, anti-thyroglobulin; Tg, thyroglobulin; TRAb, thyroid stimulating hormone receptor antibody; rT3, reverse T3; iPTH, intact parathyroid hormone.
